# Foodborne norovirus outbreak: the role of an asymptomatic food handler

**DOI:** 10.1186/1471-2334-10-269

**Published:** 2010-09-15

**Authors:** Irene Barrabeig, Ariadna Rovira, Javier Buesa, Rosa Bartolomé, Rosa Pintó, Hortènsia Prellezo, Àngela Domínguez

**Affiliations:** 1 Epidemiological Surveillance Unit of Costa Ponent. Directorate of Public Health. Department of Health, Autonomous Government of Catalonia, Barcelona, Spain; 2 Department of Microbiology. University of Valencia, Spain; 3 Department of Microbiology. Vall d'Hebron University Hospital. Barcelona, Spain; 4 Enteric Virus Laboratory. University of Barcelona, Spain; 5 Health Protection Agency. Department of Health, Barcelona, Spain; 6 Department of Public Health, School of Medicine, University of Barcelona, Spain; 7 CIBER Epidemiología y Salud Pública (CIBERESP), Instituto Carlos III. Madrid, Spain

## Abstract

**Background:**

In July 2005 an outbreak of acute gastroenteritis occurred on a residential summer camp in the province of Barcelona (northeast of Spain). Forty-four people were affected among residents and employees. All of them had in common a meal at lunch time on 13 July (paella, round of beef and fruit). The aim of this study was to investigate a foodborne norovirus outbreak that occurred in the residential summer camp and in which the implication of a food handler was demonstrated by laboratory tests.

**Methods:**

A retrospective cohort study was designed. Personal or telephone interview was carried out to collect demographic, clinical and microbiological data of the exposed people, as well as food consumption in the suspected lunch. Food handlers of the mentioned summer camp were interviewed.

Ten stool samples were requested from symptomatic exposed residents and the three food handlers that prepared the suspected food. Stools were tested for bacteries and noroviruses. Norovirus was detected using RT-PCR and sequence analysis.

Attack rate, relative risks (RR) and its 95% confidence intervals (CI) were calculated to assess the association between food consumption and disease.

**Results:**

The global attack rate of the outbreak was 55%. The main symptoms were abdominal pain (90%), nausea (85%), vomiting (70%) and diarrhoea (42.5%). The disease remitted in 24-48 hours. Norovirus was detected in seven faecal samples, one of them was from an asymptomatic food handler who had not eaten the suspected food (round of beef), but cooked and served the lunch. Analysis of the two suspected foods isolated no pathogenic bacteria and detected no viruses. Molecular analysis showed that the viral strain was the same in ill patients and in the asymptomatic food handler (genotype GII.2 Melksham-like).

**Conclusions:**

In outbreaks of foodborne disease, the search for viruses in affected patients and all food handlers, even in those that are asymptomatic, is essential. Health education of food handlers with respect to hand washing should be promoted.

## Background

The consumption of food contaminated by microorganisms is a common cause of gastroenteritis outbreaks in developed countries. Historically, bacteria have been the most-frequent causal agents, although the causal microorganism is not identified on many occasions [1-3].

Norovirus, a member of the *Caliciviridae *family, is considered the major cause of acute gastroenteritis in all age groups worldwide [4]. The majority of human noroviruses can be classified into two genogroups, I (GI) and II (GII), which are subdivided into genotypes (at least 15 GI and 18 GII). Molecular epidemiological studies suggest that a number of norovirus genotypes circulate in a variety of settings. Genogroup II, genotype 4 (GII.4) has been the most prevalent genotype in many countries in recent years [1,5].

Foods may be contaminated by contact with human faecal matter at the source [6] or by unhygienic manipulation by a food handler excreting the virus [4], although this second scenario is probably underestimated because it is difficult to prove [1].

Unlike bacteria, which multiply very easily, viruses are very difficult to detect in foods. Therefore, virological study of stool samples and epidemiological analysis of patients are the only ways of identifying the outbreak in the majority of cases. The virus can be excreted in the absence of symptoms [7], but incorrect manipulation by a food handler is necessary to produce an outbreak in which the food handler is involved.

The objective of this study was to investigate the epidemiological characteristics of an outbreak of gastroenteritis due to norovirus that occurred in a residential summer camp in July 2005 and in which the involvement of a food handler was demonstrated.

## Methods

On 15 July 2005, an outbreak of infectious gastroenteritis was detected at a residential summer camp in the province of Barcelona (Catalonia) in the northeast of Spain. Eighty-nine people were in the summer camp: 72 children aged 9-13 years, 8 monitors, 6 employees and 3 food handlers.

During the night of 14/15 July, fifteen children presented acute gastroenteritis with a predominance of nausea, vomiting and abdominal pain. The existence of cases in the community was discarded after consultation with local physicians.

The water of the summer camp was supplied directly from the municipal network. The free residual chlorine level was 0.3 mg/l in samples taken from the summer camp's water pipes.

Preparation of the meals consumed was reviewed by personal interview with the three food handlers who had prepared them. One food handler (FH A) presented symptoms of acute gastroenteritis around 16.30 hours on 14 July, while the other two (FH B and C) were asymptomatic. No family members of the food handlers were affected.

The initial interview showed that the only meal eaten by all persons affected was lunch on 13 July, which consisted of paella, round of beef and fruit. Two (FH A and B) of the three food handlers had consumed some of the suspected food: one had gastrointestinal symptoms (FH A) and the other was asymptomatic (FH B). The third (FH C) ate at her home.

### Epidemiological investigation

It was hypothesized that the outbreak of gastroenteritis was foodborne and a retrospective cohort study was designed to verify the hypothesis.

An exposed person was defined as someone who ate paella and/or round of beef for lunch on 13 July at the summer camp; fresh fruit was not considered suspicious as it was peeled by each consumer. A case was defined as an exposed person who presented vomiting or diarrhoea (three or more loose stools within 24 h) and at least two of the following symptoms: nausea, abdominal pain or fever measured by thermometer (≥37.8°C).

Personal or telephone interview was carried out to collect information on: 1. individual demographic characteristics such as sex, age, occupation; 2. suspected food consumed at lunch, 3. if ill, type, data of onset of symptoms, clinical course of the disease and family history of acute gastroenteritis.

### Laboratory investigation

Stool samples were requested from symptomatic exposed persons and the three food handlers.

Stool cultures were carried out to isolate enteropathogens (*Salmonella*, *Shigella*, *Yersinia*, *Vibrio*, *Campylobacter*, *Aeromonas *and Shiga toxin-producing strains of *Escherichia coli*). Noroviruses were detected by reverse transcription polymerase chain reaction (RT-PCR).

RT-PCR: Primers designed for partial RNA polymerase region (ORF1) amplification were used: NVp110 (5'-ACD ATY TCA TCA TCA CCA TA-3') for reverse transcription and JV12 (5'-ATA CCA CTA TGA TGC AGA TTA-3') and JV13 (5'-TCA TCA TCA CCA TGA AAA GAC-3') for PCR. Norovirus genotyping was performed by sequencing the 326-nucleotide amplimers with the JV12 and JV13 primers. Prior to phylogenetic analyses, translated amino acid sequences were aligned using the Clustal-X program with the default parameters. Using this alignment as a template, nucleotide sequences were aligned with the GeneDoc program. A phylogenetic tree was constructed by the UPGMA clustering method of Molecular Evolutionary Genetics Analysis (MEGA version 3.1).

A sample of each of the two suspected foods from the lunch on 13 July (paella and round of beef) was sent to the laboratory for analysis.

The data in this study is the result of the epidemiological and virological investigation performed by the Department of Health's corresponding to the Epidemiological Surveillance Unit of the Costa de Ponent region where the outbreak took place. Basic data (type of outbreak, date of outbreak onset, number of affected, attack rate, confirmed agent responsible for the outbreak, food vehicle) are publically available in the Outbreak summary issue of the Epidemiological Butlletin of Catalonia (BEC) [8]. This investigation was exempted from the ethical committee approval because was part of the interventions of the national public health to control outbreaks.

### Statistical Analysis

Proportions were compared using the χ^2 ^test or Fisher's exact test (when indicated). Means were compared using the Student's t test. The level of statistical significance was established as an alpha error of 0.05.

Relative risks (RR) and its 95% confidence intervals (CI) were calculated to assess the association between food consumption and disease.

Data collected were analyzed using Microsoft Access and the statistical analysis was made with the Epidat and SPSS/PC v.15 statistical packages.

## Results

Of the 85 people exposed to lunch on 13 July 2005, 80 were interviewed: 67 children (5 children exposed could not be located), 8 monitors and 5 camp workers, including two of the food handlers (FH A and B).

Forty-four people fulfilled the case definition, with a median age of 11 years (range: 9 - 50 years): 29 (66%) were male. The global attack rate was 55% (44/80), and was 48.5% in females and 59.2% in males, with no differences according to age and sex. The onset of symptoms for children, monitors and camp workers occurred between 14 and 17 July 2005, with a peak on 14 July (59% of cases).

**Figure 1:**
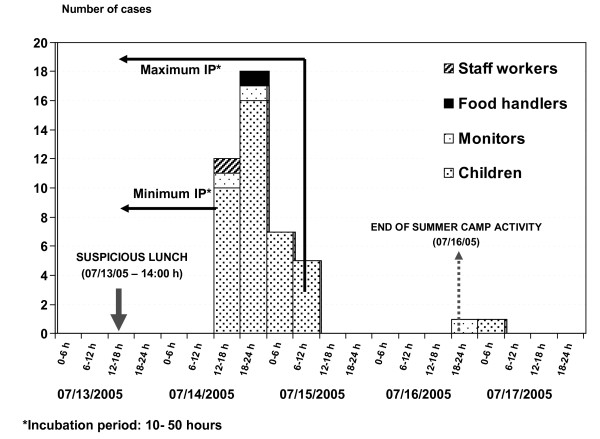
**Distribution of patients with acute gastroenteritis according to date of onset of symptoms (July 2005)**.

The epidemic curve of the first 42 cases corresponded to a single, one-time exposure. When the incubation period of norovirus was considered and the minimum incubation period (10 hours) was subtracted from the first cases and the maximum period (50 hours) from the cases on the 15 July, this showed that the suspected exposure was the lunch on 13 July (Figure 1).

The incubation period of the first cases, considering the day and hour of the suspected exposure (14.00 hours on 13 July), was 25-44.5 hours (mean: 32 hours), which was compatible with norovirus. If the source of infection of the two last cases was the suspected food, the incubation period would be 78 and 83 hours, respectively; however these cases were probably due to person-to-person transmission, and so were excluded from the food exposure analysis.

For all cases, the main symptoms were abdominal pain (90%), nausea (85%), vomiting (70%) and diarrhoea (42.5%). Of cases with fever (27.5%), none had a temperature >38°C. No difference was observed in the clinical pattern according to gender. No case required hospital admission. The disease remitted in 24-48 hours.

In the ten stool samples analyzed (nine children and one monitor), no bacterial enteropathogens were found, but norovirus was detected by RT-PCR in six samples. Norovirus was also detected in one food handler (FH B), who had no gastroenteritis.

Of the two food handlers exposed to the suspected foods, FH A had eaten round of beef and presented clinical symptoms around 16.30 hours on 14 July (Figure 1). FH B had only eaten paella and did not present symptoms. When re-surveyed, FH B stated that there had been no cases of gastroenteritis in her family and there were no symptoms of gastroenteritis two months before or after the suspected lunch.

**Figure 2:**
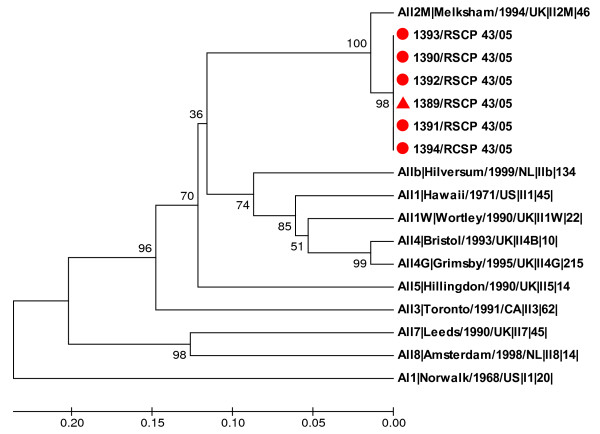
**Phylogenetic tree of partial RNA polymerase sequences of the viral strains detected in the food handler and five cases, and reference strains**. The tree was constructed using the UPGMA clustering method. Bootstrap values (1,000 replicates) are shown at the branch nodes. The food handler's sample is labeled with a triangle and the five patient specimens are labeled with round circles. The sequences of reference strains are from the Foodborne Viruses in Europe database http://www.eufoodborneviruses.co.uk/.

The norovirus strain implicated in the outbreak was identified as genotype GGII.2 Melksham-like by sequencing a portion of the RNA polymerase gene of the viral RNA extracted from specimens from patients and the food handler B (Figure 2).

In the analysis of the two suspected food, pathogenic bacteria was not isolated and virus were not detected.

Of the foods implicated, 73 people ate paella, 38 of whom became ill (attack rate: 52%) and 76 people ate round of beef, 42 of whom became ill (attack rate: 55.3%). Statistical analysis of the two suspected foods showed that neither was significantly associated with the disease (Table 1).

**Table 1:** Attack rates, attributable risks and relative risks for specific foods.

	*Exposed*			*Not exposed*				
				
Food	Ill (n)	Not ill (n)	Attack Rate (%)	Ill (n)	Not ill (n)	Attack Rate (%)	Attributable Risk	Relative Risk (95% CI)
Paella	38	35	52.0	4	1	80.0	-28.0	0.65 (0.39 - 1.06)
Round of Beef	42	34	55.3	0	2	0.0	55.3	3.31* (0.26 - 41,92)

July 2005.

*0.5 is added to all frequencies.

## Discussion

The epidemiological and clinical features identified (symptoms, incubation period, disease duration), together with the laboratory results, were consistent with the initial hypothesis of a foodborne norovirus outbreak, fulfilling Kaplan criteria [9].

As in other outbreaks [10], the statistical analysis did not establish any food as the vehicle of infection, because almost all diners had eaten all the foods. However, two facts suggest that the food vehicle was probably the round of beef (Table 1). Firstly, everyone with symptoms of gastroenteritis had eaten round of beef (both paella and round of beef or only beef). Secondly, the four people who became ill after eating beef but not paella were: a child in whom norovirus was detected, the symptomatic food hander (FH A) and two children from whom clinical samples could not be obtained.

Although laboratory analysis of the suspected foods did not find norovirus, this in no way discards the possible implication of the round of beef, since it is known that, in foods other than shellfish, the sensitivity of the analysis is very low due to the low levels of the virus normally present in contaminated foods, but enough to cause infection, and to the presence of RT-PCR inhibitors [2,6,11,12].

The most important finding of the investigation was the presence of norovirus in stool samples from an asymptomatic food handler (FH B), who denied having had symptoms before or after the suspected lunch. She had not consumed the suspected round of beef, but she had prepared, cut and served it to the diners (including FH A). Although vomiting may be a source of infection [13,14], this possibility may be discarded, as very little more than 24 hours had passed between the appearance of the first cases and the taking of stool samples from FH B. Phylogenetic analysis confirmed that the sequence of the virus detected in FH B was identical to that detected in cases (Genotype GII.2). The region of the genome chosen for sequencing (the RNA-dependent RNA polymerase) is regarded as a good target to discriminate different norovirus genotypes, although it has also been reported that the hypervariable region encoding the P2 domain in the capsid gene can be helpful in tracking transmission events within outbreaks [15]. Unlike GII.4, GII.2 is not a prevalent genotype, meaning that a possible chance association could be ruled out. In addition, children and monitors lived in different towns than FH B. Therefore, our results suggest that the source of the infection was probably an asymptomatic food handler.

Asymptomatic excretion of norovirus is recognized in facilities with outbreaks [16,17] and without outbreaks [18]. Ozawa *et al*. showed that the prevalence of norovirus detection in food handlers was 19% in different food-catering settings in Japan, and that detection of norovirus in asymptomatic food handlers associated with outbreaks was 7%, a substantial proportion.

Transmission by a food handler may be difficult to prove and there is little information about their implication in the transmission of the virus, even though it has been shown that GII-norovirus infected asymptomatic individuals have mean viral loads similar to those of symptomatic subjects (3.31 × 10^8 ^versus 5.53 × 10^8 ^copies/g of stool), showing the importance of transmission by people who are infected but not ill [17-19]. In addition, a very low dose of virus is needed to infect (10-100 viral particles). Although there are not sufficient studies to show the role of asymptomatic food handlers, our study suggests that asymptomatic carriers may be infectious.

Daniels *et al*. were the first authors to demonstrate the implication of an asymptomatic food handler in an outbreak [20], when they found the same genomic sequence of norovirus in the implicated food and in relatives of a food handler not exposed to the food. Molecular analysis of the genomic sequences of the virus detected in the food and in food handlers would be desirable in this kind of outbreak, but due to the difficulty in detecting the virus in foods the association is usually established either by identifying the same viral genome in the food handler and in affected people or through analytical epidemiological studies. Therefore, stool samples from affected people are essential since it allows the common origin of the cases to be shown. Simultaneously, stool samples from all food handlers should be analyzed, together with the epidemiological information available. Molecular epidemiology analysis of norovirus detected in patients and food handlers should be extended when epidemiological information suggests that the source may be a food handler.

Monitoring of safe practices in the food chain [9,12,21,22] and the recommendation that food handlers with diarrhoea should not work until 48-72 hours after becoming asymptomatic [7-23] are the current strategies employed to prevent foodborne infections. Several studies have demonstrated that viral shedding of norovirus may last longer [24-27]. In one study in which the virus was inoculated in fifty volunteers, the results showed that specimens collected 7 days after inoculation remained positive [24]. In another human experimental norovirus infection model, the virus was detected in stool samples for a median of 4 weeks and for up to 8 weeks after virus inoculation [27]. Although virus detection in faeces does not mean the virus is infectious, the low infective dose and prolonged shedding of norovirus makes transmission almost certain. Therefore, the recommendation of exclusion of workers for 48-72 hours after disappearance of symptoms may not be sufficient to avoid the transmission of infection.

Moreover, a recent study evaluating the effectiveness of control measures during a norovirus outbreak showed that the implementation of recommended strategies reduced transmission of the virus by 85% (95% predictive interval: 81-87%), but could not contain the outbreak [28]. The use of protective barriers such as gloves, aprons, facemasks and hairnets seems not to be effective against norovirus.

In an investigation of a gastroenteritis outbreak, accurate diagnosis and early identification of the origin of contaminated food is essential to prevent new cases, since it enables control measures to be implemented. However, it is not established which measures should be introduced - hand washing, use of barriers or correct cleaning of environmental surfaces [2,7]. At present, we do not know how to stop norovirus transmission. Strategies focus on correct hand washing as a preventive action and assessing the efficacy of disinfectants in eliminating the virus from the environment (water and surfaces). Effective prevention strategies should be determined and implemented according to scientific evidence.

## Conclusions

The results of this study show that, in outbreaks of norovirus gastroenteritis, analysis of stool samples from all food handlers (symptomatic and asymptomatic) is necessary. Given the importance of food handlers in the prevention of norovirus infections, all means should be used to encourage health education on hand washing, the hygienic manipulation of food, cleaning and disinfection.

## Abbreviations

FH: Food handler; RT-PCR: Reverse transcription-polymerase chain reaction; RR: Relative risks; CI: Confidence Interval

## Competing interests

The authors declare that they have no competing interests.

## Authors' contributions

IB and AD designed the study and drafted the manuscript. JB, RB and RP performed the microbiological analysis. IB and AR participated in the acquisition of outbreak data and performed the statistical analysis. HP participated in the environmental investigation. All authors have read and approved the final manuscript.

## Pre-publication history

The pre-publication history for this paper can be accessed here:

http://www.biomedcentral.com/1471-2334/10/269/prepub
